# PAQR3 inhibits proliferation and aggravates ferroptosis in acute lymphoblastic leukemia through modulation Nrf2 stability

**DOI:** 10.1002/iid3.437

**Published:** 2021-05-06

**Authors:** Ling Jin, Laigen Tong

**Affiliations:** ^1^ Department of Hematology Yixing People's Hospital Yixing City Jiangsu Province China

**Keywords:** ALL, ferroptosis, Nrf2, PAQR3, proliferation

## Abstract

**Introduction:**

Acute lymphoblastic leukemia (ALL) is a usual hematological tumor, which was featured by malignant proliferation of lymphoid progenitor cells. Many important factors participate into the regulation of ALL, including proteins. PAQR3 (also named RKTG) has been proved to take part in many human cancers by acting as a tumor suppressor. PAQR3 has bee n shown to repress human leukemia cells proliferation and induce cell apoptosis, but its role and relevant regulatory mechanism on cell proliferation and ferroptosis in ALL needs more exploration.

**Methods:**

The genes expression was detected through quantitative reverse transcription polymerase chain reaction (mRNA) or western blot (protein). The cell proliferation was assessed through Cell Counting Kit‐8 and 5‐ethynyl‐2‐deoxyuridine assays. The levels of MDA, DCF, and intracellular free Fe in ALL cells were tested through the commercial kits. The cell apoptosis was determined through flow cytometry analysis. The binding ability of PAQR3 and nuclear factor erythroid 2‐related factor 2 (Nrf2) was verified through pull down assay.

**Results:**

PAQR3 expression was firstly assessed in ALL patients and cell lines, and discovered to be downregulated. It was verified that PAQR3 suppressed ALL cells proliferation. Further experiments proved that PAQR3 aggravates ferroptosis in ALL. In addition, AQR3 bound with Nrf2, and modulated its expression through ubiquitination in ALL. Finally, through rescue assays, it was demonstrated that Nrf2 overexpression reversed the effects of PAQR3 on cell proliferation and ferroptosis.

**Conclusion:**

Findings from our work uncovered that PAQR3 inhibited proliferation and aggravated ferroptosis in ALL through modulation Nrf2 stability. This study suggested that PAQR3 may serve as an effective biological marker for ALL treatment.

## Graphical abstract

PAQR3 expression was downregulated in ALL PAQR3 suppressed ALL cells proliferation.

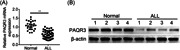

## 1 INTRODUCTION

Acute lymphoblastic leukemia (ALL) is defined as a malignant tumor of the hematopoietic system that characterized by abnormal proliferation of lymphoid or myeloid progenitors, and frequently occurred in the youth as well as seriously affected their lives.^1^, ^2^ The occurrence of ALL is due to the accumulation of genetic and epigenetic abnormalities, which lead to the changes of cell differentiation, apoptosis, proliferation, and self‐renewal ability.^3^, ^4^ For now, the 5‐year survival rate in ALL patients is unsatisfactory.^5^ Research in the past decades have discovered a large number of genetic factors, which participated into the ALL development and progression.^6^, ^7^ However, the molecular biomarkers involved in ALL progression are not well exploited. Further investigations on the molecular biological targets and their relevant mechanisms in ALL are conducive to the exploration of new therapeutic methods, improvement of prognosis, and discovery of new prognostic indicators.

More and more evidence illustrated that alterations in genes can affect the malignant behaviors of ALL cells. For example, Yin‐Yang‐1 retards cell apoptosis induced by Fas in ALL under hypoxic conditions.^8^ Additionally, SOCS3 methylation regulated JAK/STAT pathway to affect the progression of pediatric ALL.^9^ GAS2 activated Wnt/β‐Catenin pathway in pediatric T‐cell ALL to facilitate cell proliferation and repress apoptosis.^10^ Besides, PAX5‐ELN oncoprotein accelerates multistep B‐cell ALL in mice.^11^ The Progestin and AdipoQ Receptor (PAQR) family is made up of different types of membrane receptors.^12^ PAQR3 is one member of the PAQR family, a newly discovered tumor suppressor, and takes part in a variety of biological processes.^13^, ^14^ For instance, PAQR3 modulates the NF‐κB/p53/Bax axis to suppress non‐small‐cell lung cancer progression.^15^ Furthermore, PAQR3 suppresses glioma progression by inactivating PI3K/Akt pathway.^16^ MiR‐15b/PAQR3 axis contributes to breast cancer progression.^17^ In addition, PAQR3 overexpression suppresses ERK pathway to weaken the development of esophageal squamous cell carcinoma.^18^ In addition, one study has revealed that PAQR3 inhibits proliferation and facilitates apoptosis of leukemic cells (U937).^19^ However, the role and related regulatory mechanism of PAQR3 in ALL remain still unclear.

In present study, it was focused on the role of PAQR3 and its effects on cell proliferation and ferroptosis in ALL. Findings from this study demonstrated that PAQR3 exhibited lower expression in ALL patients and cell lines. In the end, we proved that PAQR3 inhibited proliferation and aggravated ferroptosis in ALL through modulation nuclear factor erythroid 2‐related factor 2 (Nrf2) stability. This discovery suggested that there was a novel potential treatment factor implicated in ALL.

## 2 MATERIALS AND METHODS

### 2.1 Samples

Samples collected from Yixing People's Hospital are used in this study, there are 25 healthy volunteers and 43 patients with ALL. All the patients had received no treatment before surgery, and signed informed consents. Blood was acquired from both ALL patients and healthy volunteers. T cells were isolated through the EasySep human T cell isolation kit (STEMCELL Technologies). These samples were frozen in liquid‐nitrogen and then stored at −80°C until further experiments. Our work was ratified by the Ethics Committee of Yixing People's Hospital.

### 2.2 Cell culture and transfection

Human acute lymphoblastic leukemia cell lines (MOLT3, MOLT4, CEM‐C1, and Jurkat) were brought from the American Type Culture Collection (ATCC). These cells were cultured in RPMI‐1640 medium (Invitrogen) including 10% FBS (Invitrogen) plus 1% penicillin/streptomycin. They were maintained in a humidified incubator with 5% CO_2_ at 37°C. Cycloheximide (CHX) was applied to examine the protein degradation of Nrf2 at 0, 2, 4, and 8 h. Erastin as ferroptosis inducer was used to treat ALL cells for 24 h in concentrations of 0–50 μM; RSL3 as ferroptosis inducer was used to treat ALL cells for 24 h in concentrations of 0–0.5 μM; Fer‐1 as ferroptosis inhibitor was used to treat ALL cells in the concentration of 1 μM.

The pcDNA3.1/PAQR3 vectors were constructed by cloning its full‐length into the pcDNA3.1 vectors (Thermo Fisher Scientific), and pcDNA3.1/Nrf2 vectors were constructed as the same way, with pcDNA3.1 vectors as the control. The transfection for these vectors was performed through Lipofectamine 2000 (Invitrogen).

### 2.3 Quantitative reverse transcription polymerase chain reaction (RT‐qPCR)

RNAs were extracted from ALL samples or cells using Trizol reagent (Invitrogen). Synthesis of cDNA was done by the PrimeScript® RT reagent Kit (Takara). The expression of genes was detected by SYBR Green PCR kit (TaKaRa) using GAPDH as endogenous controls. The data were processed using the $2^{‐∆∆C_{t}}$  method. All primers were as follows:

PAQR3:

F: 5′‐TGTCGAAGATGGATGGCATTAGA‐3′;

R: 5′‐ACCTGACGCCAGTAGTATTACACACA‐3′.

NQO1:

F: 5′‐ACTCGGAGAACTTTCAGTACC‐3′;

R: 5′‐TTGGAGCAAAGTAGAGTGGT‐3′.

HO‐1:

F: 5′‐ATCGTGCTCGCATGAACACT‐3′;

R: 5′‐CCAACACTGCATTTACATGGC‐3′.

GCLC:

F: 5′‐TTAGGCTGTCCTGGGTTCAC‐3′;

R: 5′‐TCGCTCCTCCCGAGTTCTAT‐3′.

FTH1:

F: 5′‐AAGCTGCAGAACCAACGAGG‐3′;

R: 5′‐AGTCACACAAATGGGGGTCATT‐3′.

GAPDH:

F: 5′‐ACAGTCAGCCGCATCTTCT‐3′;

R: 5′‐GACAAGCTTCCCGTTCTCAG‐3′.

### 2.4 CCK‐8 assay

Cell viability was tested through CCK‐8 (Dojindo). In short, transfected CEM‐C1 and Jurkat cells were placed on 96‐well plates at a density of 1 × 10^4^ cells/well. Each well was added with 10 μl of CCK‐8 reagent and then incubated for another 4 h. The cell viability was evaluated at the absorbance of 450 nm by using a Microplate Reader (Bio‐Rad).

### 2.5 5‐Ethynyl‐2‐deoxyuridine (EdU) assay

This assay was carried out through the EdU incorporation assay kit (RiboBio). Transfected ALL cells were inoculated into 96‐well plates and then cultivated with 50 µM EdU labeling medium for 2 h. Subsequently, cells were fixed in 4% paraformaldehyde, next incubated with 0.5% Troxin X‐100 and then treated with 1× Apollo® reaction cocktail. 4′,6‐Diamidino‐2‐phenylindole was applied to stain the nuclei and the images were obtained through the fluorescent microscope (Olympus).

### 2.6 Detection of MDA, DCF, and Fe

The levels of reactive oxygen species (ROS), MDA, and Fe^2+^ in ALL cells were detected through DCF ROS Assay Kit (ab238535; Abcam), Lipid peroxidative Assay Kit (ab118970; Abcam), and Iron Assay Kit (ab83366; Abcam).

### 2.7 Flow cytometry assay

Cell apoptosis was assessed through the Annexin V‐FITC Apoptosis Detection Kit (Abcam). Generally, after washing twice with cold PBS solution, CEM‐C1, and Jurkat cells were resuspended. Afterward, Annexin V‐FITC and propidium iodide were added and incubated for 10 min in the dark. Finally, the apoptosis rate was tested under a flow cytometer (BD Biosciences).

### 2.8 Coimmunoprecipitation (co‐IP) analysis

Briefly, cells were transfected with Flag‐tagged Nrf2 or Myc‐tagged PAQR3, and then the proteins were isolated. These proteins were incubated with anti‐Flag (ab205606; Abcam), anti‐Myc (ab32; Abcam) at 4°C for overnight. Protein A‐agarose beads (Cell Signaling Technology) were then added and incubated for another 4 h. After washing three times, bound proteins were measured through western blot.

### 2.9 Western blot assay

ALL samples or cells were lysed through RIPA lysis buffer (Beyotime). Proteins were subjected to 10% sodium dodecyl sulfate polyacrylamide gel electrophoresis and then passed onto polyvinylidene difluoride membranes. After being blocked with 5%‐skim milk, membranes were incubated with primary antibodies: anti‐PAQR3 (1:1000; ab174327; Abcam), anti‐Nrf2 (1:1000; ab137550; Abcam) and anti‐β‐actin (1:1000; ab8226; Abcam) overnight at 4°C. Next, membranes were incubated with HRP‐conjugated secondary antibodies. Bands were observed by the ECL chemiluminescent detection system (Thermo Fisher Scientific).

### 2.10 Statistical analysis

Statistical analysis was done through SPSS 20.0 (SPSS, Inc.). The data were showed as the mean ± *SD*. Statistical differences between two groups (or among multiple groups) were carried out through Student's *t* test (or one‐way analysis of variance). *p* < .05 was supposed as statistically significant.

## 3 RESULTS

### 3.1 PAQR3 expression was downregulated in ALL

**Figure 1:**
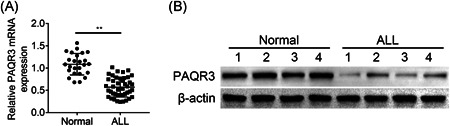
PAQR3 expression was downregulated in ALL. (A) The PAQR3 mRNA expression was tested through RT‐qPCR in healthy volunteers (*n* = 25) and ALL patients (*n* = 43). (B) The PAQR3 protein expression was examined through western blot in the normal group (*n* = 4) and the ALL group (*n* = 4). **p < .01. ALL, acute lymphoblastic leukemia; mRNA, messenger RNA; RT‐qPCR, quantitative reverse transcription polymerase chain reaction

The healthy volunteers (*n* = 25) and ALL patients (*n* = 43) were recruited to detect PAQR3 expression in our work. Results from RT‐qPCR illustrated that compared with the Normal group, PAQR3 exhibited lower mRNA expression in ALL patients' T cell (Figure 1A). Furthermore, we discovered that the protein expression of PAQR3 was downregulated in ALL patients compared with the normal volunteers (Figure 1B). Taken together, PAQR3 expression was downregulated in ALL.

### 3.2 PAQR3 suppressed ALL cells proliferation

**Figure 2:**
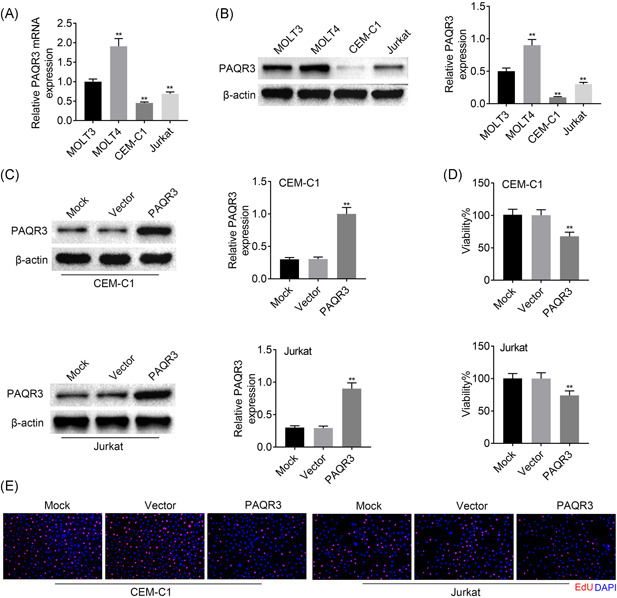
PAQR3 suppressed ALL cells proliferation. (A) The PAQR3 mRNA expression was measured through RT‐qPCR in MOLT3, MOLT4, CEM‐C1, and Jurkat cells. (B) The PAQR3 protein expression was detected through western blot in MOLT3, MOLT4, CEM‐C1, and Jurkat cells. (C) The overexpression efficiency of PAQR3 was verified through RT‐qPCR CEM‐C1 and Jurkat cells. (D) The cell proliferation was identified through CCK‐8 assay after overexpressing PAQR3. (E) The cell proliferation was also examined through EdU assay after PAQR3 upregulation. **p < .01. ALL, acute lymphoblastic leukemia; CCK‐8, Cell Counting Kit‐8; EdU, 5‐ethynyl‐2‐deoxyuridine; mRNA, messenger RNA; RT‐qPCR, quantitative reverse transcription polymerase chain reaction

Next, the PAQR3 mRNA and protein expression was examined in ALL cells. The mRNA and protein expression of PAQR3 was upregulated in MOLT4 cells, and downregulated in CEM‐C1 and Jurkat cells (Figure 2A,B). Due to the lower PAQR3 expression in CEM‐C1 and Jurkat cells, these two cell lines were selected for further experiments. As displayed in Figure 2C, the overexpression efficiency of PAQR3 was verified. PAQR3 protein expression was enhanced after overexpressing PAQR3 in CEM‐C1 and Jurkat cells. Then, findings from CCK‐8 assay revealed that the cell viability of CEM‐C1 and Jurkat cells was weakened after overexpressing PAQR3 (Figure 2D). In addition, the similar results were discovered in Figure 2E through EdU assay, and cell proliferation was decreased after upregulating PAQR3. These data suggested that PAQR3 suppressed ALL cells proliferation.

### 3.3 PAQR3 aggravated ferroptosis in ALL

**Figure 3:**
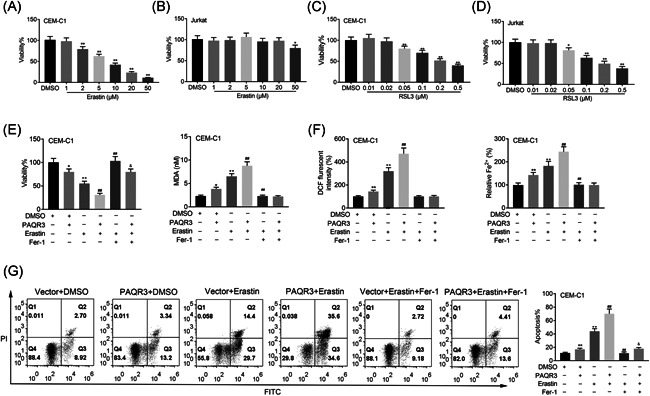
PAQR3 aggravates ferroptosis in ALL. (A) The cell viability was confirmed through CCK‐8 assay in CEM‐C1 and Jurkat cells treated with Erastin. (B) The cell viability was measured through CCK‐8 assay in CEM‐C1 cells and Jurkat cells treated with RSL3. (C) The cell viability of CEM‐C1 cells was verified through CCK‐8 assay in the Vector + DMSO, PAQR3 + DMSO, Vector + Erastin, PAQR3 + Erastin, Vector + Erastin + Fer‐1, and PAQR3 + Erastin + Fer‐1 groups. (D–F) The levels of MDA, DCF, and Fe in CEM‐C1 cells were tested through the appropriate kits in the Vector + DMSO, PAQR3 + DMSO, Vector + Erastin, PAQR3 + Erastin, Vector + Erastin + Fer‐1, and PAQR3 + Erastin + Fer‐1 groups. (G) The apoptosis rate of CEM‐C1 cells was measured through flow cytometry in the Vector + DMSO, PAQR3 + DMSO, Vector + Erastin, PAQR3 + Erastin, Vector + Erastin + Fer‐1, and PAQR3 + Erastin + Fer‐1 groups. Erastin: 10 μM; Fer‐1: 1 μM. *p < .05, **p < .01; ^#^ *p* < .05, ^##^ *p* < .01; ^&^ *p* < .05, ^&&^ *p* < .01. ALL, acute lymphoblastic leukemia; CCK‐8, Cell Counting Kit‐8; DMSO, dimethyl sulfoxide

Through next experiments, we further investigated the impacts of PAQR3 on ferroptosis in ALL. The cell viability was reduced with the increased dose of Erastin (the ferroptosis inducer) in CEM‐C1 cells. In Jurkat cells, cell viability was slightly reduced upon the administration of 50 μM Erastin, but not at lower concentrations (Figure 3A). The cell viability was weakened with the increased dose of RSL3 (the ferroptosis inducer) in both CEM‐C1 and Jurkat cells (Figure 3B). CEM‐C1 is the mutant‐type of KRAS, while Jurkat is the wild‐type of RAS. Therefore, Jurkat is not sensitive to Erastin, but Jurkat is sensitive to RSL3.^20^, ^21^ Next, the cell viability of CEM‐C1 cells was decreased after PAQR3 overexpression or Erastin treatment, and further decreased after PAQR3 overexpression plus Erastin treatment. On the base of treating with Erastin, the cell viability was enhanced by Fer‐1 (the ferroptosis inhibitor) treatment, but this effect could be offset after overexpressing PAQR3 (Figure 3C). Ferroptosis is a programmed cell death (PCD) caused by iron‐dependent lipid peroxidation (oxidative stress), so that the levels of MDA, DCF and Fe were further tested in our work. The levels of MDA, DCF, and Fe in CEM‐C1 cells were enhanced in the PAQR3 + DMSO or Vector + Erastin group, and further strengthened in the PAQR3 + Erastin group. The MDA, DCF, and Fe levels was reduced in the Vector + Erastin + Fer‐1 group compared with the Vector + Erastin group, but no prominent changes was found after PAQR3 overexpression (Figure 3D–F). The apoptosis rate of CEM‐C1 cells was increased after PAQR3 overexpression or Erastin treatment, and further aggravated after PAQR3 overexpression plus Erastin treatment. On the base of treating with Erastin, PAQR3 overexpression reversed the reduced cell apoptosis mediated by Fer‐1 treatment (Figure 3G). This finding suggested that Fer‐1 could completely reverse the cell apoptosis caused by RSL3, but it could not completely reverse the cell apoptosis caused by PAQR3, indicating that there were other pathways in cell apoptosis caused by PAQR3 besides ferroptosis.

**Figure 4:**
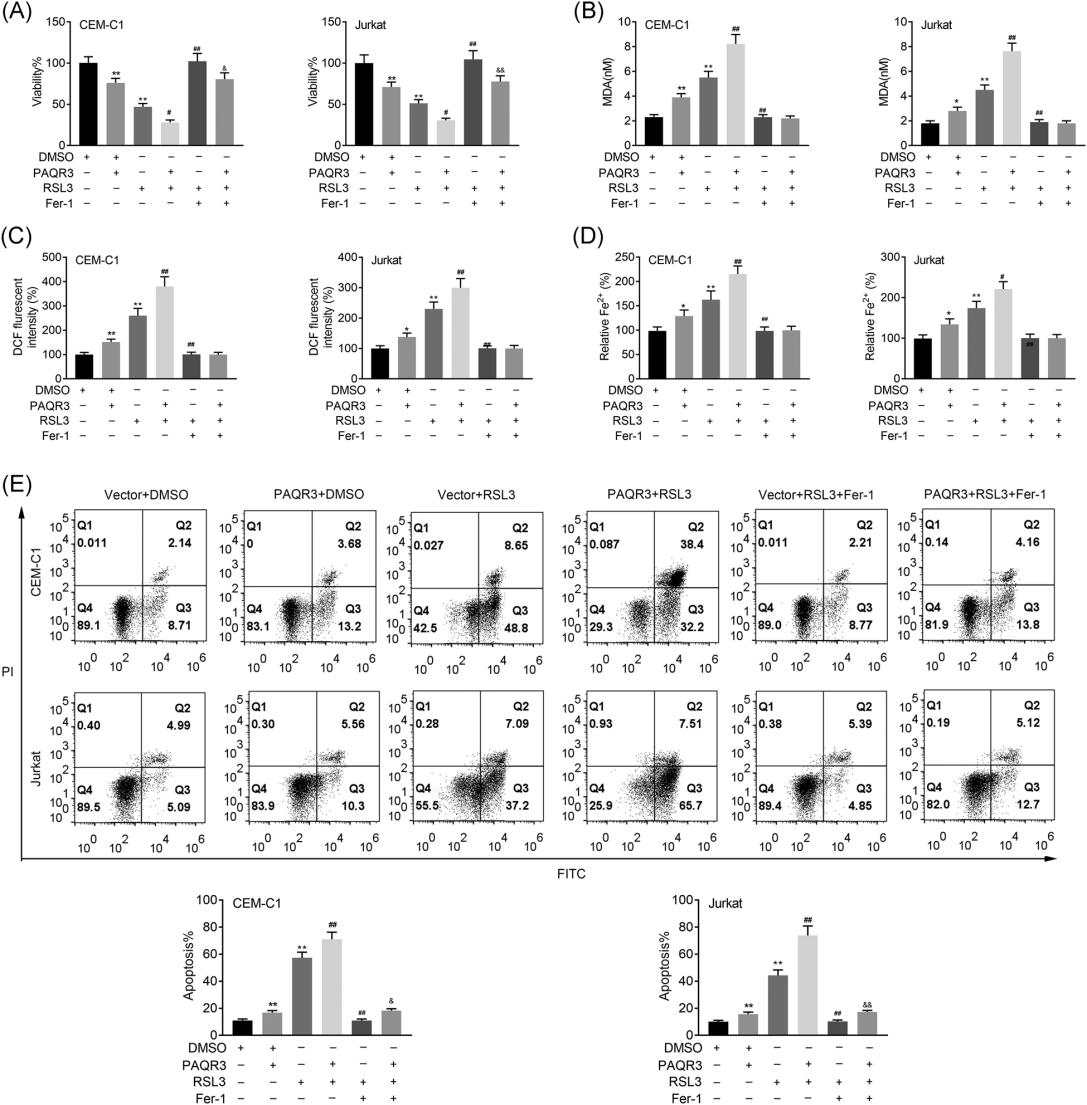
PAQR3 aggravates ferroptosis in ALL. (A) The cell viability of CEM‐C1 and Jurkat cells was verified through CCK‐8 assay in the Vector + DMSO, PAQR3 + DMSO, Vector + RSL3, PAQR3 + RSL3, Vector + RSL3 + Fer‐1, and PAQR3 + RSL3 + Fer‐1 groups. (B–D) The levels of MDA, DCF and Fe in CEM‐C1 and Jurkat cells were tested through the appropriate kits in the Vector + DMSO, PAQR3 + DMSO, Vector + RSL3, PAQR3 + RSL3, Vector + RSL3 + Fer‐1, and PAQR3 + RSL3 + Fer‐1 groups. (E) The apoptosis rate of CEM‐C1 and Jurkat cells was measured through flow cytometry in the Vector + DMSO, PAQR3 + DMSO, Vector + RSL3, PAQR3 + RSL3, Vector + RSL3 + Fer‐1, and PAQR3 + RSL3 + Fer‐1 groups. Fer‐1: 1 μM; RSL3: 0.1 μM. *p < .05, **p < .01; ^#^ *p* < .05, ^##^ *p* < .01; ^&^ *p* < .05, ^&&^ *p* < .01. ALL, acute lymphoblastic leukemia; CCK‐8, Cell Counting Kit‐8; DMSO, dimethyl sulfoxide

Additionally, the cell viability of CEM‐C1 and Jurkat cells was decreased after PAQR3 overexpression or RSL3 treatment, and further weakened after PAQR3 overexpression plus RSL3 treatment. On the base of treating with RSL3, the cell viability was enhanced by Fer‐1 treatment, but this effect could be attenuated after overexpressing PAQR3 (Figure 4A). The levels of MDA, DCF and Fe in CEM‐C1 and Jurkat cells were enhanced in the PAQR3 + DMSO or Vector + RSL3 group, and further heightened in the PAQR3 + RSL3 group. The MDA, DCF and Fe levels were reduced in the Vector + RSL3 + Fer‐1 group compared with the Vector + RSL3 group, but no prominent changes was found after PAQR3 overexpression (Figure 4B–D). The apoptosis rate of CEM‐C1 and Jurkat cells was increased after PAQR3 overexpression or RSL3 treatment, and further strengthened after PAQR3 overexpression plus RSL3 treatment. On the base of treating with RSL3, PAQR3 overexpression attenuated the reduced cell apoptosis induced by Fer‐1 treatment (Figure 4E). To sum up, PAQR3 aggravated ferroptosis in ALL.

### 3.4 PAQR3 modulated Nrf2 in ALL

**Figure 5:**
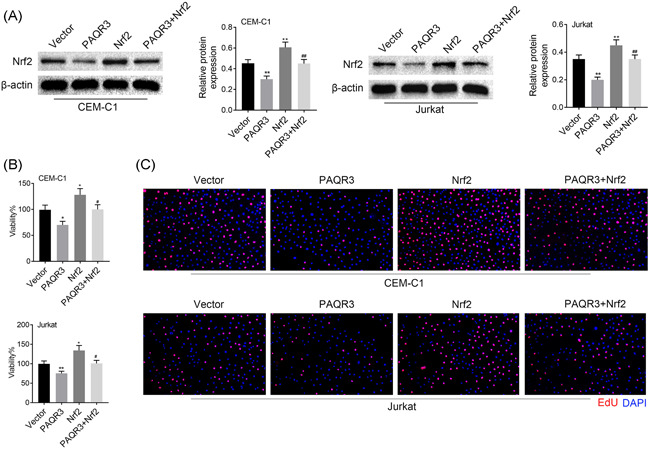
Nrf2 overexpression reversed the inhibitive effects of PAQR3 on cell proliferation. (A) The Nrf2 protein expression was detected through western blot in the Vector, PAQR3, Nrf2, and PAQR3 + Nrf2 groups. (B) The cell viability was verified through CCK‐8 assay in the Vector, PAQR3, Nrf2, and PAQR3 + Nrf2 groups. (C) The cell proliferation was measured through EdU assay in the Vector, PAQR3, Nrf2, and PAQR3 + Nrf2 groups. *p < .05, **p < .01; ^#^ *p* < .05, ^##^ *p* < .01. CCK‐8, Cell Counting Kit‐8; EdU, 5‐ethynyl‐2‐deoxyuridine

PAQR3 could regulate the degradation of Nrf2, and Nrf2 plays a regulator in ferroptosis.^22^ Therefore, we speculated whether PAQR3 regulated ferroptosis through Nrf2. The Nrf2 mRNA had no changes, but Nrf2 protein expression was downregulated after upregulating PAQR3 (Figure 5A,B). Through co‐IP assay, it was demonstrated that PAQR3 bound with Nrf2 in CEM‐C1 cells (Figure 5C). Moreover, the Nrf2 protein expression was decreased after overexpressing PARQ3 with treating with the increased treatment times of CHX, suggesting that Nrf2 protein degraded faster after PAQR3 overexpression (Figure 5D). Further exploration showed that the mRNA expression Nrf2‐related downstream genes (NQO1, HO‐1, GCLC, and FTH1) were all downregulated after PAQR3 overexpression (Figure 5E). In a word, PAQR3 modulated Nrf2 in ALL.

### 3.5 Nrf2 overexpression reversed the inhibitive effects of PAQR3 on cell proliferation

**Figure 6:**
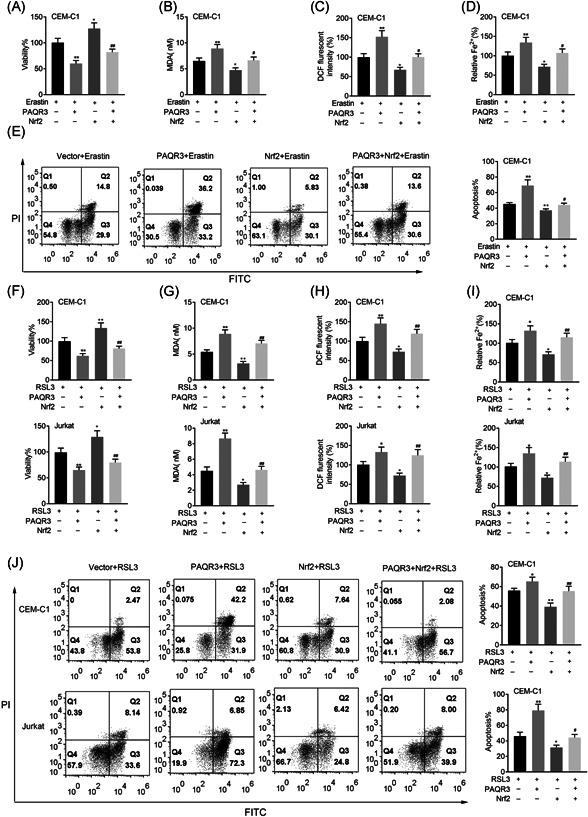
Nrf2 overexpression rescued the effects of PAQR3 on ferroptosis. (A) The cell viability of CEM‐C1 cells was inspected through CCK‐8 assay in the Vector + Erastin, PAQR3 + Erastin, Nrf2 + Erastin, and PAQR3 + Nrf2 + Erastin group. (B–D) The levels of MDA, DCF, and Fe in CEMC1 cells were measured through the appropriate kits in the Vector + Erastin, PAQR3 + Erastin, Nrf2 + Erastin, and PAQR3 + Nrf2 + Erastin group. (E) The apoptosis rate of CEM‐C1 cells was detected through flowcytometry in the Vector + Erastin, PAQR3 + Erastin, Nrf2 + Erastin, and PAQR3 + Nrf2 + Erastin group. (F) The cell viability of CEM‐C1 and Jurkat cells was inspected through CCK‐8 assay in the Vector + RSL3, PAQR3 + RSL3, Nrf2 + RSL3, and PAQR3 + RSL3 + Erastin group. (G–I) The levels of MDA, DCF, and Fe in CEM‐C1 and Jurkat cells were measured through the appropriate kits in the Vector + RSL3, PAQR3 + RSL3, Nrf2 + RSL3, and PAQR3 + RSL3 + Erastin group. (J) The apoptosis rate of CEM‐C1 and Jurkat cells was detected through flowcytometry in the Vector + RSL3, PAQR3 + RSL3, Nrf2 + RSL3, and PAQR3 + RSL3 + Erastin group. *p < .05,**p < .01; ^#^ *p* < .05, ^##^ *p* < .01. CCK‐8, Cell Counting Kit‐8

Rescue assays were performed to confirm whether Nrf2 overexpression reversed the inhibitive effects of PAQR3 on cell proliferation. Overexpression of Nrf2 rescued the downregulated Nrf2 protein level induced by PAQR3 overexpression (Figure 6A). The cell viability was decreased in the PAQR3 group compared to the Vector group, but this effect was reversed in the PAQR3 + Nrf2 group (Figure 6B). Moreover, through EdU assay, cell proliferation was weakened after overexpressing PAQR3, but this effect could be offset by Nrf2 upregulation (Figure 6C). These findings indicated that Nrf2 overexpression reversed the inhibitive effects of PAQR3 on cell proliferation.

### 3.6 Nrf2 overexpression rescued the effects of PAQR3 on ferroptosis

**Figure 7:**
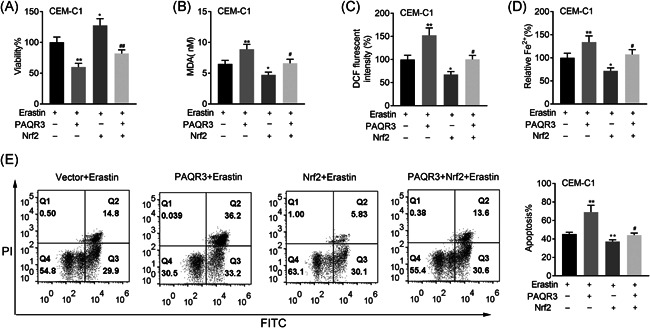
Nrf2 overexpression rescued the effects of PAQR3 on ferroptosis. (A) The cell viability of CEM‐C1 cells was inspected through CCK‐8 assay in the Vector + Erastin, PAQR3 + Erastin, Nrf2 + Erastin, and PAQR3 + Nrf2 + Erastin group. (B–D) The levels of MDA, DCF, and Fe in CEM‐C1 cells were measured through the appropriate kits in the Vector + Erastin, PAQR3 + Erastin, Nrf2 + Erastin and PAQR3 + Nrf2 + Erastin group. (E) The apoptosis rate of CEM‐C1 cells was detected through flow cytometry in the Vector + Erastin, PAQR3 + Erastin, Nrf2 + Erastin, and PAQR3 + Nrf2 + Erastin group. *p < .05, **p < .01; ^#^ *p* < .05, ^##^ *p* < .01. CCK‐8, Cell Counting Kit‐8

Rescue assays were also conducted to verify whether Nrf2 overexpression rescued the effects of PAQR3 on ferroptosis. On the treatment of Erastin, overexpression of Nrf2 could reverse the decreased cell viability induced by overexpressing PAQR3 (Figure 7A). The MDA, DCF and Fe levels were enhanced in the Erastin + PAQR3 group, but this effect was rescued in the Erastin + PAQR3 + Nrf2 group (Figure 7B–D). On the treatment of Erastin, PAQR3 overexpression reduced cell apoptosis, but this effect could be offset by upregulating Nrf2 (Figure 7E).

**Figure 8:**
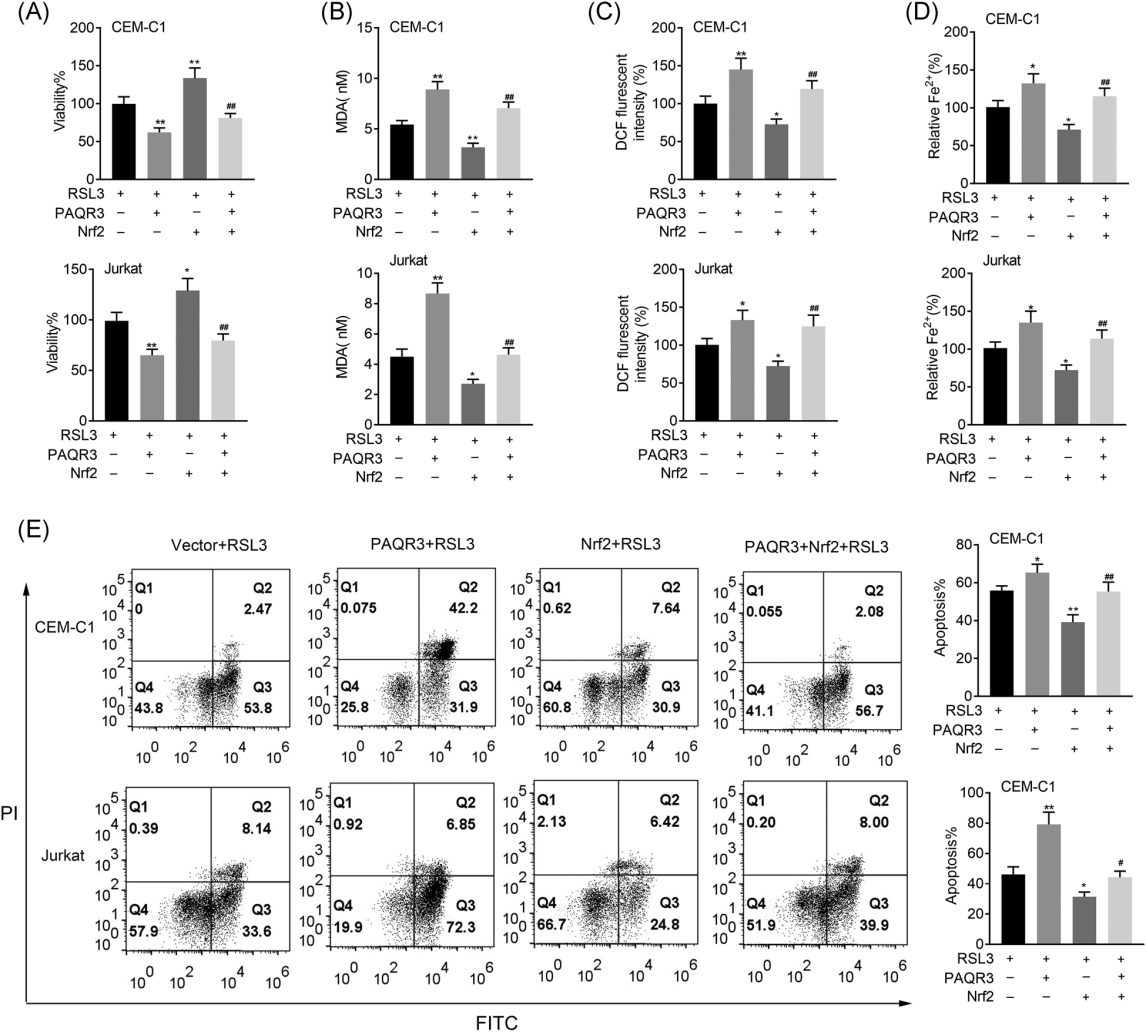
Nrf2 overexpression rescued the effects of PAQR3 on ferroptosis. (A) The cell viability of CEM‐C1 and Jurkat cells was inspected through CCK‐8 assay in the Vector + RSL3, PAQR3 + RSL3, Nrf2 + RSL3, and PAQR3 + RSL3 + Erastin group. (B–D) The levels of MDA, DCF, and Fe in CEM‐C1 and Jurkat cells were measured through the appropriate kits in the Vector + RSL3, PAQR3 + RSL3, Nrf2 + RSL3, and PAQR3 + RSL3 + Erastin group. (E) The apoptosis rate of CEM‐C1 and Jurkat cells was detected through flow cytometry in the Vector + RSL3, PAQR3 + RSL3, Nrf2 + RSL3 and PAQR3 + RSL3 + Erastin group. *p < .05, **p < .01; ^#^ *p* < .05, ^##^ *p* < .01. CCK‐8, Cell Counting Kit‐8

On the treatment of RSL3, overexpression of Nrf2 could reverse the decreased cell viability induced by overexpressing PAQR3 (Figure 8A). The MDA, DCF, and Fe levels were enhanced in the RSL3 + PAQR3 group, but this effect was rescued in the RSL3 + PAQR3 + Nrf2 group (Figure 8B–D). On the treatment of RSL3, PAQR3 overexpression reduced cell apoptosis, but this effect could be offset by upregulating Nrf2 (Figure 8E). These data manifested that Nrf2 overexpression rescued the effects of PAQR3 on ferroptosis.

## 4 DISCUSSION

With receiving more and more attention, an increasing number of key proteins have been identified in ALL.^8^, ^9^, ^10^, ^11^ PAQR3 has been uncovered to be a critical regulator into various cancers.^15^, ^16^, ^17^, ^18^ In particular, researchers found PAQR3 affect leukemic cells proliferation and apoptosis,^19^ nevertheless, its role and relevant regulatory mechanism in ALL keeps unknown. In this study, PAQR3 expression was firstly assessed in ALL, and discovered to be downregulated. Furthermore, results from CCK‐8 and EdU assays verified that PAQR3 suppressed ALL cells proliferation.

Ferroptosis is a newly discovered PCD, which is characterized by iron‐dependent peroxide aggregation.^23^ It is a new cell death mode which is different from the typical apoptosis and other PCD, and its morphological features are cell volume shrinkage and increase of mitochondrial membrane density.^24^, ^25^ Ferroptosis inducers can be divided into two types. The first type of inducers includes Erastin, sulfasalazine, and buthionine sulfoximine. These inducers inhibit system Xc^‐^ (the reverse transporter of glutamic acid and cyine) and reduce glutathione (GSH) levels in cells, thereby inducing redox imbalance.^26^ The second type of inducers includes a series of artificial compounds such as RSL3, DPI7, DPI10, DPI12, and DPI13, which directly inhibit glutathione peroxidase 4 (GPX4) and lead to the accumulation of peroxides in cells.^27^ Finally, due to the abnormal metabolism of iron ions in cells, the accumulation of ROS leads to ferroptosis.^28^ At present, there are few studies on ferroptosis in ALL. In our work, further experiments proved that PAQR3 aggravates ferroptosis in ALL cells.

The Nrf2, a transcription factor highly sensitive to oxidative stress, which combine with antioxidant response element (ARE) in the nucleus to facilitate the transcription of various antioxidant genes.^29^, ^30^ Interestingly, the two ferroptosis inducers, RSL‐3 and Erastin could inhibit GPX4 and xC^−^/xCT, and GPX4 and xC^−^/xCT are downstream targets of Nrf2.^31^ Therefore, Nrf2 has emerged as a key regulator of ferroptosis. For example, Nrf2 suppresses ferroptosis to improve acute lung injury mediated by seawater drowning.^32^ Besides, NRF2 regulates FOCAD‐FAK pathway to affect the sensitivity of NSCLC cells to ferroptosis induced by cystine deprivation.^33^ Repression of Nrf2 reverses resistance to ferroptosis induced by GPX4 inhibitor in head and neck cancer.^34^ Moreover, Upregulation of Nrf2 inhibits ferroptosis to retard diabetic nephropathy progression.^35^ PAQR3 has been reported to may exert its biological functions by interacting with other proteins. For instance, PAQR3 interacts with Sec13/Sec31 coat proteins to modulate the transportation of COPII vesicle.^36^ In addition, DDB2 interacts with PAQR3 to modulate the tumorigenesis of gastric cancer.^37^ It also has been reported that PAQR3 regulates the regulatory subunits of COMPASS‐like complexes to affect H3K4 trimethylation in mammalian cells.^38^ It was hypothesized that PAQR3 and Nrf2 may directly interact with each other. In this study, we discovered that PAQR3 bound with Nrf2, and modulated its expression through regulating stability in ALL. Additionally, PAQR3 negative regulated the downstream genes (NQO1, HO‐1, GCLC, and FTH1) of Nrf2 in ALL. Finally, through rescue assays, it was demonstrated that Nrf2 overexpression reversed the effects of PAQR3 on cell proliferation and ferroptosis.

In general, it was the first time to discover that PAQR3 inhibited proliferation and aggravated ferroptosis in ALL through modulation Nrf2 stability. This discovery may offer new sights for ALL treatment. However, our work still exists some limitations, more experiments will be done for further exploration of PAQR3 in the future.

## CONFLICT OF INTERESTS

The authors declare that there are no conflict of interests.

## AUTHOR CONTRIBUTIONS

Ling Jin and Laigen Tong designed the study and supervised the data collection. Ling Jin analyzed the data and interpreted the data. Laigen Tong prepare the manuscript for publication and reviewed the draft of the manuscript. All authors have read and approved the manuscript.

## ETHICS STATEMENT

Ethical approval was obtained from the Ethics Committee of Yixing People's Hospital.
